# Clinical Analysis of *Pseudomonas aeruginosa* Infections in Children Undergoing Chemotherapy or Hematopoietic Cell Transplantation: A Multicenter Nationwide Study

**DOI:** 10.3390/jcm14113714

**Published:** 2025-05-26

**Authors:** Monika Richert-Przygońska, Krzysztof Czyżewski, Patrycja Zalas-Więcek, Olga Gryniewicz-Kwiatkowska, Agnieszka Gietka, Zofia Małas, Katarzyna Semczuk, Liliana Chełmecka, Iwona Żak, Małgorzata Salamonowicz-Bodzioch, Jowita Frączkiewicz, Olga Zając-Spychała, Ewa Bień, Ninela Irga-Jaworska, Marcin Płonowski, Paweł Wawryków, Magdalena Bartnik, Filip Pierlejewski, Zuzanna Gamrot, Wanda Badowska, Weronika Stolpa, Jakub Musiał, Anna Szmydki-Baran, Łukasz Hutnik, Renata Tomaszewska, Agnieszka Urbanek-Dądela, Agnieszka Zaucha-Prażmo, Jolanta Goździk, Jan Styczyński

**Affiliations:** 1 Department of Pediatrics, Hematology and Oncology, Collegium Medicum, Nicolaus Copernicus University Torun, 85-094 Bydgoszcz, Poland; kczyzewski@cm.umk.pl (K.C.); jstyczynski@cm.umk.pl (J.S.); 2 Department of Microbiology, Collegium Medicum, Nicolaus Copernicus University Torun, 85-094 Bydgoszcz, Poland; 3 Department of Oncology, Children’s Memorial Health Institute, 04-730 Warsaw, Poland; o.gryniewicz@ipczd.pl (O.G.-K.); zofia.malas@imid.med.pl (Z.M.); 4 Department of Microbiology, Children’s Memorial Health Institute, 04-730 Warsaw, Poland; 5 Department of Pediatric Oncology and Hematology, University Children’s Hospital, Jagiellonian University, Collegium Medicum, 31-008 Krakow, Poland; 6 Department of Microbiology, University Children’s Hospital, 31-008 Krakow, Poland; 7 Department of Pediatric Stem Cell Transplantation, Hematology and Oncology, Medical University, 50-556 Wroclaw, Poland; 8 Department of Pediatric Oncology, Hematology and Transplantology, University of Medical Sciences, 61-701 Poznan, Poland; 9 Department of Pediatrics, Hematology and Oncology, Medical University, 80-210 Gdansk, Poland; 10 Department of Pediatric Oncology and Hematology, Medical University, 15-089 Bialystok, Poland; mar26@mp.pl; 11 Department of Pediatrics, Pediatric Oncology and Immunology, Pomeranian Medical University, 70-204 Szczecin, Poland; 12 Department of Pediatrics, Pediatric Hematooncology and Gastroenterology, Pomeranian Medical University, 70-204 Szczecin, Poland; 13 Department of Pediatrics, Hematology and Oncology, Medical University, 91-738 Lodz, Poland; 14 Division of Pediatric Hematology and Oncology, Children Hospital, 10-561 Olsztyn, Poland; 15 Division of Pediatric Oncology, Hematology and Chemotherapy, Department of Pediatric, Silesian Medical University, 40-752 Katowice, Poland; 16 Department of Pediatric Oncohematology, Medical Faculty University of Rzeszow, Clinical Provincial Hospital No. 2, 35-301 Rzeszow, Poland; 17 Department of Pediatric Hematology and Oncology, Medical University, 02-091 Warszawa, Poland; 18 Department of Pediatric Hematology and Oncology, Silesian Medical University, 41-800 Zabrze, Poland; 19 Division of Pediatric Hematology and Oncology, Children Hospital, 25-736 Kielce, Poland; 20 Department of Pediatric Hematology, Oncology and Transplantology, Medical University, 20-400 Lublin, Poland; 21 Stem Cell Transplant Center, University Children’s Hospital, Department of Clinical Immunology and Transplantology, Jagiellonian University, Collegium Medicum, 31-008 Krakow, Poland

**Keywords:** *Pseudomonas aeruginosa*, hematopoietic cell transplantation, children

## Abstract

**Background**: *Pseudomonas aeruginosa* (PSA) infections are associated with a high recurrence rate and high mortality in immuno-compromised patients. There are limited studies regarding pediatric hematopoietic cell transplantation recipients. **Aim**: The nationwide multicenter study was conducted to analyze the epidemiology of PSA infections in children treated with chemotherapy (PHO, pediatric hematology and oncology) or undergoing hematopoietic allogeneic or autologous cell transplantation (HCT) in the period 2014–2023. **Methods**: We retrospectively analyzed the clinical and microbiological data of children who underwent anticancer therapy or hematopoietic cell transplantation in 17 Polish PHO centers and six pediatric HCT centers. The data were collected in two-year intervals. **Results**: During the 10-year study period, a total of 1629 HCTs (both autologous and allogeneic) and 9614 children newly diagnosed with neoplasms were analyzed. The cumulative incidence of PSA infection was similar in both groups (6.71% in PHO vs. 6.32% in HCT, *p* = 0.624). The total number of PSA bloodstream infections was comparable in the PHO and HCT groups (31.9% vs. 26.2%; *p* = 0.223). In both analyzed groups, the antipseudomonal drugs of choice were as follows: meropenem, ceftazidime, and tazobactam/piperaciline in combination with other antibiotics. In the HCT group, high rates of meropenem (20.4%) and tazobactam/piperaciline (18.4%) non-susceptibility were observed. This led to colistin therapy in 5.3% of patients. There was no difference in the median antibiotic therapy time in both groups; however, the survival rates from PSA infection were significantly lower in the HCT group (89.3% vs. 96.0%, *p* = 0.004). **Conclusions**: Although the risk of infection and the occurrence of resistant bacterial strains in HCT patients were comparable with those in PHO patients, the outcome of PSA infections was better in the PHO setting.

## 1. Introduction

Bacterial infections, especially those caused by Gram-negative rods, are one of the main clinical situations triggering infection-related mortality in neutropenic patients [1,2]. *Pseudomonas aeruginosa* (PSA) is one of the most life-threatening pathogens among hospitalized, immunocompromised patients during cancer therapy, radiotherapy, and for those suffering from malnutrition, burns, or severe external injuries, as well as those undergoing medical procedures such as catheterization, surgery, or hematopoietic cell transplantation (HCT). Invasive PSA infections are characterized by a high recurrence and mortality rate [3]. The major factors of PSA pathogenicity are as follows: wide genome plasticity, hypermutable strains, an ability to form biofilm, numerous intrinsic virulence determinants such as secreting systems, toxin production, and inactivating enzymes, and finally, molecular antibiotic resistance mechanisms [4,5]. Possible clinical manifestations of PSA infection include pneumonia, urinary tract infections, skin and soft tissue infections, keratitis, endocarditis, bacteremia, or sepsis in a high percentage of hematological patients. The mortality rate of PSA infections is a huge concern, especially in the case of carbapenem-resistant strains or multidrug-resistant (MDR) isolates [6,7,8]. The limited data available indicate that severe PSA infections in the pediatric population are associated with mortality due to the incidence of community-acquired and hospital-acquired PSA infections being much higher than in adults, as well as the inappropriate use of empiric therapy or sepsis [9,10,11].

Although many treatment regimens and new drugs have recently been developed, widespread PSA antibiotic resistance is globally emerging [12,13]. To address PSA resistance increases and changes in PSA infection epidemiology, the World Health Organization (WHO) placed PSA on the priority list to highlight the need for a reevaluation of PSA-prevention strategies, the appropriate use of antibiotics, and likewise, the urgent need for research initiatives regarding new drugs.

The aim of the study was to analyze the cumulative incidence of PSA infection and identify the risk factors influencing drug resistance and PSA-related mortality in children undergoing chemotherapy or hematopoietic cell transplantation.

## 2. Materials and Methods

### 2.1. Study Design

We conducted the multicenter, nationwide study by analyzing the epidemiology, clinical presentation, and treatment outcomes of PSA infections in pediatric patients undergoing oncological treatment or transplant procedures. The microbiological data were evaluated with a focus on drug susceptibility.

### 2.2. Patients and Data Collection

Retrospective analysis was conducted, including on both the microbiological and clinical data of children who received chemotherapy or hematopoietic cell transplantation between 2014 and 2023. The data were gathered from 17 pediatric hematology and oncology centers across Poland, along with 6 pediatric HCT centers. The information was collected in two-year intervals to ensure comprehensive data analysis (2014–2015, 2016–2017, 2018–2019, 2020–2021, and 2022–2023).

### 2.3. Definitions

We evaluated microbiologically documented PSA infections (MDIs) only. These were defined according to the Infectious Diseases Working Party of European Society of Blood and Marrow Transplantation’s definitions. MDIs were categorized into the following: bloodstream infections, urinary tract infections, gastrointestinal infections, respiratory tract infections, ear infections, eye infections, and skin or soft tissue infections, with a separate category of wounds in the inguinal or groin area. PSA colonization was excluded from the study. Several patients undergoing the transplant procedure had a documented and ongoing PSA infection at admission.

Bloodstream infections (BSIs), urinary tract infections (UTIs), and gastrointestinal tract infections (GTIs) were defined as previously described [14]. Respiratory tract infections were identified based on the detection of viable bacteria in bronchoalveolar lavage or sputum specimen. Eye or ear infections were classified by the detection of bacteria in swab samples or wound cultures. Microbiological media and the tests used for strain identification were unified and used as previously described [15].

Infections were compared between patients of pediatric hematology and oncology centers and transplant centers. The time to development of the PSA infection was defined as the time from the diagnosis of oncological disease to the onset of infection for the PHO settings, and for the HCT settings, as the time from transplantation to infection. Antibiotic treatment for PSA was maintained until symptom resolution and microbiological eradication were achieved.

Bacterial isolates that were non-susceptible to at least one of the antipseudomonal carbapenems (meropenem, darapenem, or imipenem) were defined as carbapenem-resistant (CR) PSA. The multi-drug resistant (MDR) PSA category was defined when an isolate was non-susceptible to at least 1 agent in 3 or more antimicrobial categories. Being extensively drug resistant (XDR) was defined as PSA non-susceptible to almost all drugs in almost all categories; pandrug-resistant bacteria were defined as non-susceptible to all antipseudomonal agents [16]. Drug sensitivity tests were standardized and interpreted as was described in a previous analysis [15].

### 2.4. Antimicrobial Prophylaxis

A standardized prophylactic regimen was implemented for all neutropenic patients, following consistent protocols across all participating centers, as was outlined in previous studies [14].

### 2.5. Statistics

The cumulative incidence of PSA infection was determined using competing risk analysis, starting from the day of diagnosis (PHO setting) and from the day of transplantation (HCT setting). Death was considered a competing event. The Kaplan–Meier method was used to determine infection-free survival. Multivariate logistic regression analysis was performed for infection-free survival after infection with PSA. The hazard ratio (HR) and 95% confidence intervals (95% CI) were calculated for the difference in PSA infections between groups of patients. Competing risk analysis was based on the Fine–Gray model. The subdistribution hazard ratio in univariate and multivariate analysis accounted for death as a competing event. A *p*-value < 0.05 was considered statistically significant.

## 3. Results

During the 10-year study period, 9614 pediatric patients with newly diagnosed cancer received anticancer treatment across 17 PHO centers, while 1629 children underwent either allogeneic or autologous hematopoietic cell transplantation at six transplant centers. PSA infections were identified in 645 PHO patients and 103 HCT patients.

### 3.1. Demographics

Patients’ characteristics (N = 748) are presented in Table 1. Briefly, PHO patients were treated for acute lymphoblastic leukemia (ALL, N = 247), acute myeloblastic leukemia (AML, N = 68), Non-Hodgkin lymphoma (NHL, N = 51), Hodgkin disease (HD, N = 14), or solid tumors, such as neuroblastoma (N = 46), nephroblastoma (N = 19), rhabdomyosarcoma (N = 32), or bone tumors (N = 19).

Out of 103 HCT patients, the most common diagnoses included ALL (N = 25), AML (N = 17), aplastic anemias (AA, N = 17), myelodysplastic syndromes (N = 4), or primary immune deficiencies (N = 12).

The median age at diagnosis was 5.6 years in the PHO group, and the mean age at transplantation in the HCT group was 6.9 years. The gender distribution was similar in both groups (Table 1). The median time to neutrophil engraftment in HCT patients was 16 days (IQR 14–22). The median follow-up was 18 months (IQR 10–22) for PHO patients and 12 months (IQR 6–17) for HCT patients.

### 3.2. Cumulative Incidence of Infection

The median time to development of PSA infection was 3.9 months for PHO patients and 0.6 months for HCT patients. In the HCT group, 9/103 of patients were admitted with ongoing and already treated PSA infection.

**Figure 1:**
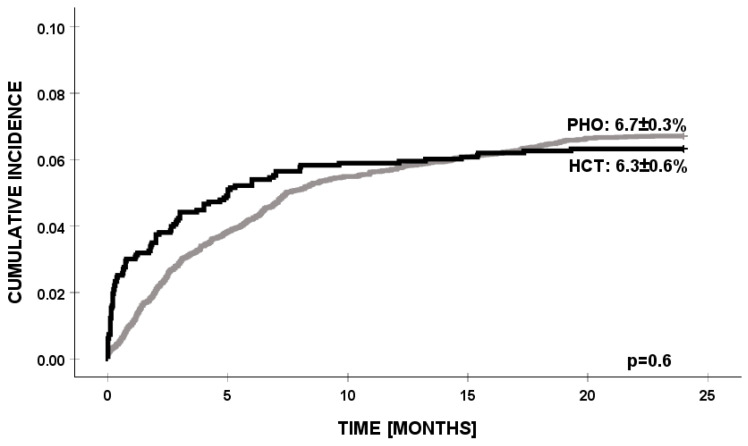
Cumulative incidence of PSA infection in PHO and HCT settings. PSA—*Pseudomonas aeruginosa*, PHO—pediatric hematology and oncology, and HCT—hematopoietic cell transplantation.

By accounting for competing events, we found that the cumulative incidence of PSA infection was significantly higher in HCT patients below 10 years of age (*p* = 0.003). In the PHO group, PSA infections were identified significantly earlier, while in the HCT group, the time from the day of transplant to PSA infection was over 5 months (*p* = 0.000). There were no other significant demographic or clinical factors affecting the cumulative incidence of PSA infection in any of the patients’ settings (Table 2). There were no differences in the cumulative incidence of infection with PSA between the PHO and HCT patients (6.71% vs. 6.32, *p* = 0.624) (Figure 1).

### 3.3. Clinical Manifestation

In PHO patients, UTIs were the most frequently documented PSA infections (N = 226). Subsequently, the next most common were BSIs (N = 196) and skin or soft tissue infections (N = 88), with a high number of patients presenting PSA inflammatory infiltrates in inguinal, groin, or perianal tissues (N = 20). Similarly, in the HCT group, the most frequently documented infections were UTIs (N = 39) and BSIs (N = 38). The total number of PSA bloodstream infections was comparable in the PHO and HCT groups (196/645 vs. 38/103; *p* = 0.223). Among the 748 patients diagnosed with PSA infection, 35 had microbiologically confirmed respiratory tract infections. For the remaining patients, positive cultures were obtained from stool samples or ear or eye swabs (Table 1).

### 3.4. Antibiotic Susceptibility and Treatment Outcomes

All patients with MDIs received antipseudomonal antibiotic treatment.

**Figure 2:**
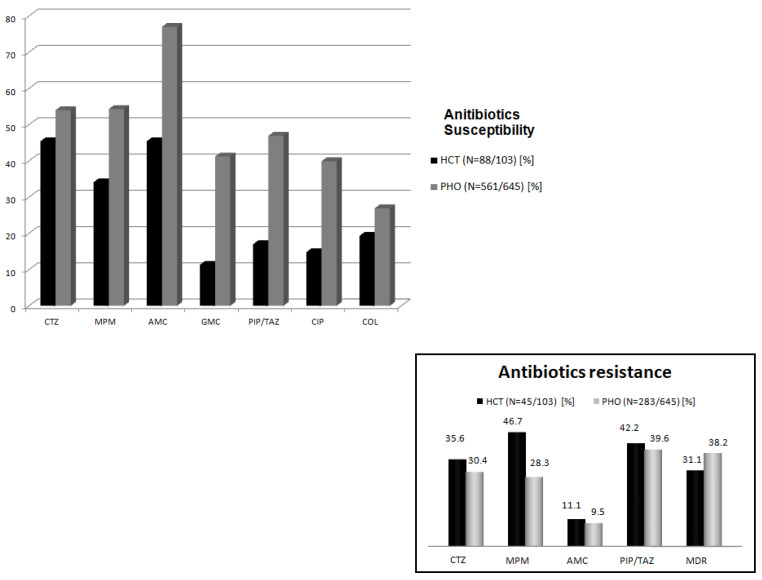
Antibiotics’ susceptibility/resistance. PHO—pediatric hematology and oncology, HCT—hematopoietic cell transplantation, CTZ—ceftazidime, MPM—meropenem, AMC—amikacin, GMC—gentamycin, PIP/TAZ—piperacilline/tazobactam, CIP—ciprofloxacin, COL—colistin, and MDR—multidrug resistance.

Among all the documented cases, susceptibility to ceftazidime was noted in 45.8%, to meropenem in 45.2%, to amikacin in 63.1%, and to piperaciline/tazobactam (PIP/TAZ) in 31.2% of strains, respectively (Figure 2).

On the contrary, a large number of non-susceptible cases were documented in both groups (291/641 in PHO; 45/103 in HCT). In the HCT setting, MDR cases were comparable with the PHO group (38.2% vs. 31.1%, *p* = 0.715). Meropenem resistance was noted in 46.7% (21/45), PIP/TAZ resistance in 42.2% (19/45), ceftazidime resistance in 35.6% (16/45), and amikacin resistance in 11.1% (5/45) of cases. Contrastingly, in the PHO group, non-susceptibility to PIP/TAZ was the most common (39.5%, 112/283) (Figure 2).

For the majority of the cases in both settings, meropenem or ceftazidime were the drugs of choice as a part of multidrug regimen. In the PHO setting, antipseudomonal monotherapy was used in 18.9% of cases, based on ceftazidime in 52 cases and meropenem in 35 cases. In the HCT group, single-drug therapy was applied in 23 cases. Among all patients, 5.3% received colistin and 2.3% were treated with an alternative drug such as ceftazidime/avibactam (N = 11); cefoperazone/sulbactam (N = 3); or aztreonam/avibactam (N = 3). The median duration of antibiotic treatment was 11 days for both groups (IQR 8–20 for HCT; IQR 9–22 for PHO patients).

**Figure 3:**
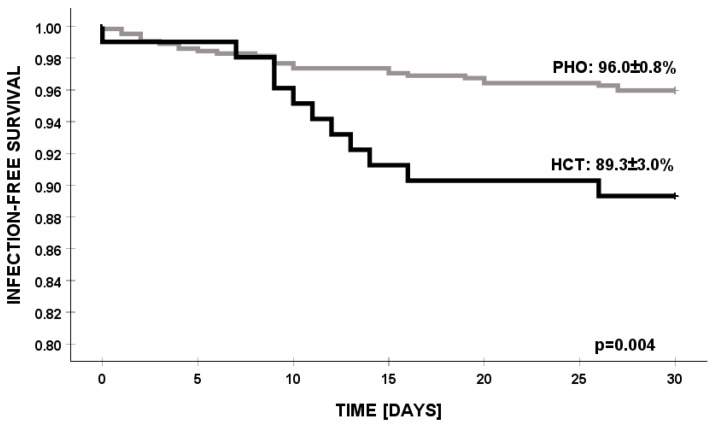
Infection-free survival of PSA infections in PHO and HCT settings. PSA—*Pseudomonas aeruginosa*, PHO—pediatric hematology and oncology, and HCT—hematopoietic cell transplantation.

The 30-day survival rate for PSA infection in the PHO setting was significantly higher compared to in the HCT group (96% vs. 89.3%, *p* = 0.004) (Figure 3).

Deaths caused by PSA infection occurred in 37/748 (4.9%) patients, mostly due to sepsis, multiorgan failure, disseminated soft tissue, or skin involvement, whereas 73 patients died because of disease progression or relapse, fungal coinfection, intracranial hemorrhage, gastrointestinal bleeding, or graft versus host disease complications.

## 4. Discussion

This nationwide multicenter study evaluated the cumulative incidence, clinical manifestations, and outcomes of PSA infection in pediatric cancer patients undergoing anticancer therapy or hematopoietic cell transplantation. Our findings suggest that while PSA infections are a significant concern for both PHO and HCT patients, the incidence, clinical presentation, and treatment outcomes exhibit some differences between these two patient cohorts.

The cumulative incidence of PSA infection was similar in both groups (PHO 6.71% vs. HCT 6.32%, *p* = 0.624), indicating that both populations are at comparable risk of PSA infections. Referring to previously published data, the cumulative incidence of PSA infection is still noticeably less frequent in our patient groups. In the adult population, PSA infection episodes are identified in 9–26% of HCT recipients and in over 20% of cases in hematological malignancies or solid tumors [1,6,13,17,18,19]. Existing pediatric data account for the MDR PSA strains for 30% of the total of 12 unselected pediatric hematology centers [20].

However, several limitations should be noted. The median follow-up of 12 moths may limit the detection of late-onset infections or long-term sequelae, potentially underestimating the true burden of disease in our study. Furthermore, heterogeneity in clinical management, including antimicrobial use and infection surveillance, may affect the comparability of results across institutions. Future studies with longer follow-up periods could provide more comprehensive insights.

Despite demographic differences in the analyzed patient cohorts and the large proportion of patients with hematologic cancers and primary immune deficiencies in the HCT group, none of these factors significantly influenced the cumulative incidence of PSA infection. The high incidence of PSA infection in the HCT group at time of admission (8.7% of patients with ongoing or treated infections) confirms that prior immunosuppressive therapies, intense and complicated treatment, or prior exposure to antipseudomonal drugs may influence PSA morbidity in both the pre- and post-transplant periods [7,13,18,21].

Most of the published data focus on BSI and septic shock manifestation, indicating PSA BSI as an independent risk factor for mortality in the pre- and post-engraftment periods [6,8]. Surprisingly in both the PHO and HCT groups, the most common manifestation of PSA infection was UTIs, followed by BSIs. These findings are consistent with the known pathophysiology of PSA infections, which commonly colonizes mucosal surfaces like the urinary tract and is a known cause of nosocomial bloodstream infections [5,22]. Interestingly, we also observed a relatively high incidence of soft tissue infection, including skin and soft tissue abscesses, which may reflect the local invasion mechanisms of PSA in immunocompromised patients. We reported 37 deaths in patients with BSI or septic shock PSA infection manifestations. Compared with previous studies, the total mortality rate (4.9%) was lower in our analysis, which may result from the differences in the local epidemiology, the specificity of pediatric patients, or the appropriate empiric therapy use and timing [6,8,17,19]. Moreover, there are several studies emphasizing the role of inappropriate initial antibiotic therapy and delayed treatment of the underlying disease, with a special focus on lung disease and respiratory difficulties as mortality risk factors. These data are even less optimistic for patients with MDR PSA infections [20,23].

PSA antibiotic resistance was a significant concern in both analyzed groups. Non-susceptibility to meropenem and piperacillin/tazobactam was higher in the HCT group, whilst antibiotic prophylaxis was unified and there were no differences in antibiotic stewardship practices. Our data differ from those previously published, in which non-susceptibilities to cefepime, ceftazidime, gentamicin, or carbapenems were the most common and most challenging [10,23]. Despite the rational guidance on the management of PSA infection from the last several years, the prevalence of carbapenem-resistant (CRP) or MDR PSA strains remains at a high level in general [24].

In our study, drug resistance resulted in colistin therapy in 5.3% of cases and the use of alternative antipseudomonal drugs in 2.3% of cases. The extremely high burden of MDR strains in both cohorts suggests that novel therapies and combination regimens will be necessary to optimize patient outcomes [6,21,24,25]. Several studies showed the promising results of ceftazidime–avibactam and ceftolozane–tazobactam activity against CRP; however, these data should be interpreted and used with caution [26,27,28]. We believe that identifying patients at risk of PSA BSI and MDR PSA is essential in selected pediatric patient groups, particularly HCT recipients.

In conclusion, our findings highlight that *Pseudomonas aeruginosa* can lead to severe infectious complications in pediatric immunocompromised patients. While the incidence of PSA infection, the presence of antibiotic-resistant strains, and invasive bloodstream infection rates among all HCT recipients were similar to those in PHO patients, the treatment outcomes for PSA infections were more favorable in the PHO cohort.

## Figures and Tables

**Table 1:** Patients’ characteristics.

PHO Centers (N = 645)		HCT Centers (N = 103)	
Diagnosis		Diagnosis	
ALL	247 (38.3%)	ALL	25 (24.3%)
AML	68 (10.5%)	AML	17 (16.5%)
Brain tumors	78 (12.1%)	AA/BMF	21 (20.4%)
NHL	51 (7.9%)	NBL	15 (14.6%)
HD	14 (2.2%)	PID	12 (11.6%)
RMS	32 (4.9%)	Others	13 (12.6%)
WT	19 (2.9%)		
NBL	46 (7.1%)		
Others	90 (14.1%)		
Age		Age	
(years/median/IQR)	5.6 (12.2–131)	(years/median/IQR)	6.8 (3.6–11.2)
Gender		Gender	
Female	313 (48.5%)	Female	47 (45.7%)
Male	332 (51.5%)	Male	56 (54.3%)
Source of infection		Source of infection	
BSI	196	BSI	38
UTI	226	UTI	39
Skin	88	Skin	14
Inguinal/perianal	20	Inguinal/perianal	
BAL	31	BAL	1
GIT	42	Eye	4
Eye	2		2
Ear	9		
Time to PSA identification (months/median/IQR)	3.9 (2.1–9.5)	Time to PSA identification (months/median/IQR)	0.6 (0–4.1)

PHO—pediatric hematology and oncology, HCT—hematopoietic cell transplantation, ALL—acute lymphoblastic leukemia; AML—acute myeloblastic leukemia; NHL—Non-Hodgkin lymphoma; HD—Hodgkin disease; RMS—rhabdomyosarcoma; WT—Wilms tumor; NBL—neuroblastoma; AA—aplastic anemia; BMF—bone marrow failure; PID—primary immunodeficiency; BSI—bloodstream infection; UTI—urinary tract infection; GIT—gastrointestinal tract infection; BAL—bronchoalveolar lavage; PSA—*Pseudomonas aeruginosa*; and IQR—interquartile range.

**Table 2:** PSA infection incidence and risk factors.

Risk Factor	Univariate Analysis (Fine–Gray Model)		Multivariate Analysis (Fine–Gray Model)	
	Subdistribution Hazard (95% CI)	*p*	Subdistribution Hazard (95% CI)	*p*
Acute leukemia vs. other diagnosis	1.20 (1.05–1.38)	0.012	1.15 (1.02–1.32)	0.021
Female vs. male	1.05 (0.91–1.20)	0.515	1.02 (0.87–1.18)	0.814
Age < 10 years vs. ≥10 years	0.45 (0.27–0.75)	**0.003**	0.48 (0.30–0.77)	**0.002**
Time to infection * <5 months vs. ≥5 months	12.6 (7.2–21.4)	**<0.001**	12.1 (6.7–21.0)	**<0.001**

PSA—*Pseudomonas aeruginosa*; * time to infection: time from start of chemotherapy in PHO group or day of transplant in HCT group, respectively; CI—confidence interval, and *p*—statistical significance.

## Data Availability

The original contributions presented in the study are included in the article. Further inquiries can be directed to the corresponding author.
